# Body shape and risk of glaucoma: A Mendelian randomization

**DOI:** 10.3389/fmed.2022.999974

**Published:** 2022-09-23

**Authors:** Ruolan Yuan, Kangcheng Liu, Yingjun Cai, Fei He, Xiaoxiong Xiao, Jing Zou

**Affiliations:** ^1^Eye Center of Xiangya Hospital, Hunan Key Laboratory of Ophthalmology, Central South University, Changsha, China; ^2^National Clinical Research Center for Geriatric Disorders, Xiangya Hospital, Central South University, Changsha, China; ^3^Department of Thoracic Surgery, Xiangya Hospital, Central South University, Changsha, China; ^4^Jiangxi Clinical Research Center for Ophthalmic Disease, Jiangxi Research Institute of Ophthalmology and Visual Science, Affiliated Eye Hospital of Nanchang University, Nanchang, China; ^5^The First Affiliated Hospital of Nanchang University, Nanchang, China

**Keywords:** body shape, glaucoma, body mass index, waist-to-hip ratio adjusted by body mass index, waist-to-hip ratio, waist circumference, Mendelian randomization

## Abstract

**Background:**

Body size (BS) is one of the risk factors for the development of many clinical diseases, but the relationship between BS and glaucoma is controversial. Herein, we try to use Mendelian randomization (MR) method to study BS causal association with glaucoma risk from the genetic level.

**Methods:**

The Body Size was determined through anthropometric traits (ATs), such as body mass index (BMI), waist-to-hip ratio adjusted by body mass index (WHRadjBMI), waist-to-hip ratio (WHR), and waist circumference (WC). Association of single nucleotide polymorphisms (SNPs) with each AT and glaucoma were determined individually from the aggregated data of the Genetic Investigation of Anthropometric Traits (GIANT) consortium and the FinnGen study summary data (8,591 cases with glaucoma and 210,201 controls). To explore the role of BS and glaucoma, a two-sample MR analysis was performed on genome-wide association study (GWAS) data. Besides, three MR methods [inverse variance weighted (IVW), Weighted median, and MR-Egger regression] were used to get the whole causal estimate for multiple instrumental SNPs.

**Results:**

BMI (OR = 1.20; 95% CI = 1.02–1.41; *P* = 0.03) and WC (OR = 1.32; 95% CI =1.04–1.69; *P* = 0.03) were associated with a risk of glaucoma. Besides, genetically predicted WHRadjBMI (OR = 1.10; 95% CI = 0.88–1.35; *P* = 0.43) and WHR (OR = 1.22; 95% CI = 0.93–1,572; *P* = 0.14) were not associated with glaucoma. No heterogeneity and directional pleiotropy were detected.

**Conclusion:**

The data of this study revealed that increased BMI and WC are potential risk factors for glaucoma, and WHRadjBMI and WHR are not associated with the occurrence of glaucoma.

## Introduction

Glaucoma is a chronic condition of progressive optic neuropathy associated with characteristic damage to the optic nerve, loss of visual field, and lead irreversible blindness. The cases of glaucoma are expected to observe an increment of 111.8 million by 2040 (1). The raised intraocular pressure (IOP) on the optic nerve is the sole modifiable risk factor in glaucoma; however, it does not help in all cases (2, 3). Thus, we need to start looking at factors other than intraocular pressure that may be associated with glaucoma to find a new method of prevention and treatment. Glaucoma is considered a multifactorial disease, and family history of glaucoma (4) and age (5) are considered the chief risk factors for glaucoma. At present, a large number of clinical studies have shown that immune components are also involved in the neurodegenerative process of glaucoma (6). In addition, other factors, including hemodynamic factors, metabolic syndrome, and obesity, were associated with glaucoma (7).

Body size (BS) is generally measured through anthropometric characteristics (ATs), like body mass index (BMI), waist to hip ratio adjusted according to body mass index (WHRadjBMI), waist to hip ratio (WHR), and waist circumference (WC) (8). Among them, BMI is a common tool to assess obesity. The World Health Organization (WHO) defines obesity as a body mass index ≥30 kg/m^2^, overweight as a body mass index between 25 to 29.9 kg/m^2^ (9). Obesity is a growing problem worldwide and has an impact on eye diseases including age-related cataracts, age-related macular degeneration and diabetic retinopathy (10). However, whether there is a direct link between obesity and intraocular pressure remains elusive. On the one hand, BMI had a positive linear correlation with IOP (11). And on the other hand, BMI was inversely associated with the risk of open-angle glaucoma (12). Whereas, no significant differences were found in BMI when comparing patients with and without glaucoma in a case-control study (13). Recently, many scholars studied the impact of anthropometric parameters on the incidence of glaucoma. In a national health and nutrition survey from South Korea, fat mass/weight ratio and fat mass/muscle mass ratio were found to be negatively associated with glaucoma. On the contrary, muscle mass parameter/BMI ratio was observed to be significantly positively related with glaucoma (*P* < 0.05) in males. In contrast, height and fat mass/BMI showed a serious relationship with onset of glaucoma (*P* < 0.05) in females (14). Furthermore, a positive correlation was found for BMI with IOP in the Chinese and Singaporeans population. (15). Conversely, lower BMI led to higher prevalence of glaucoma in the Indian population (16). Therefore, whether these parameters are positively or negatively correlated with the disorder is debatable because of ethnic differences and confounding factors found in different studies and different research methods. Although previous studies had suggested that some of the relevant items in BS may be associated with the occurrence of glaucoma, limited follow-up and research methods have made it difficult to complete randomized controlled trials to explore the specific relationship between patients with glaucoma and BS.

The Mendelian randomization (MR) analysis is a type of instrumental variable (IV)-based study, which is extensively utilized to assess potential causal relationships among exposure and outcome (17). Moreover, the gene distribution of human genetics follows Mendelian genetic law and does not get affected by most acquired confounding factors. Recently, Robert Carreras-Torres used the MR method to verify a strong causal relationship between BMI, as one of BS, and the risk of pancreatic cancer [ODDS Ratio (OR) = 1.34, 95% Confidence Interval (CI) = 1.09–1.65, for Each Standard Deviation Increase in BMI (4.6 kg/m^2^)] (18). However, as we know, there were no studies conducted using genetic data to explore the causal relationship between BS and glaucoma. Therefore, we have conducted an extensive MR analysis to understand the influence of Body Shape on glaucoma incidence by simulating the possible effects of BMI, WHRadjBMI, WHR and WC on glaucoma risk. Our results indicate the potential risks of glaucoma in BMI and WC in BS and provides new diagnostic ideas and preventive measures for glaucoma.

## Materials and methods

### Study design

**Figure 1:**
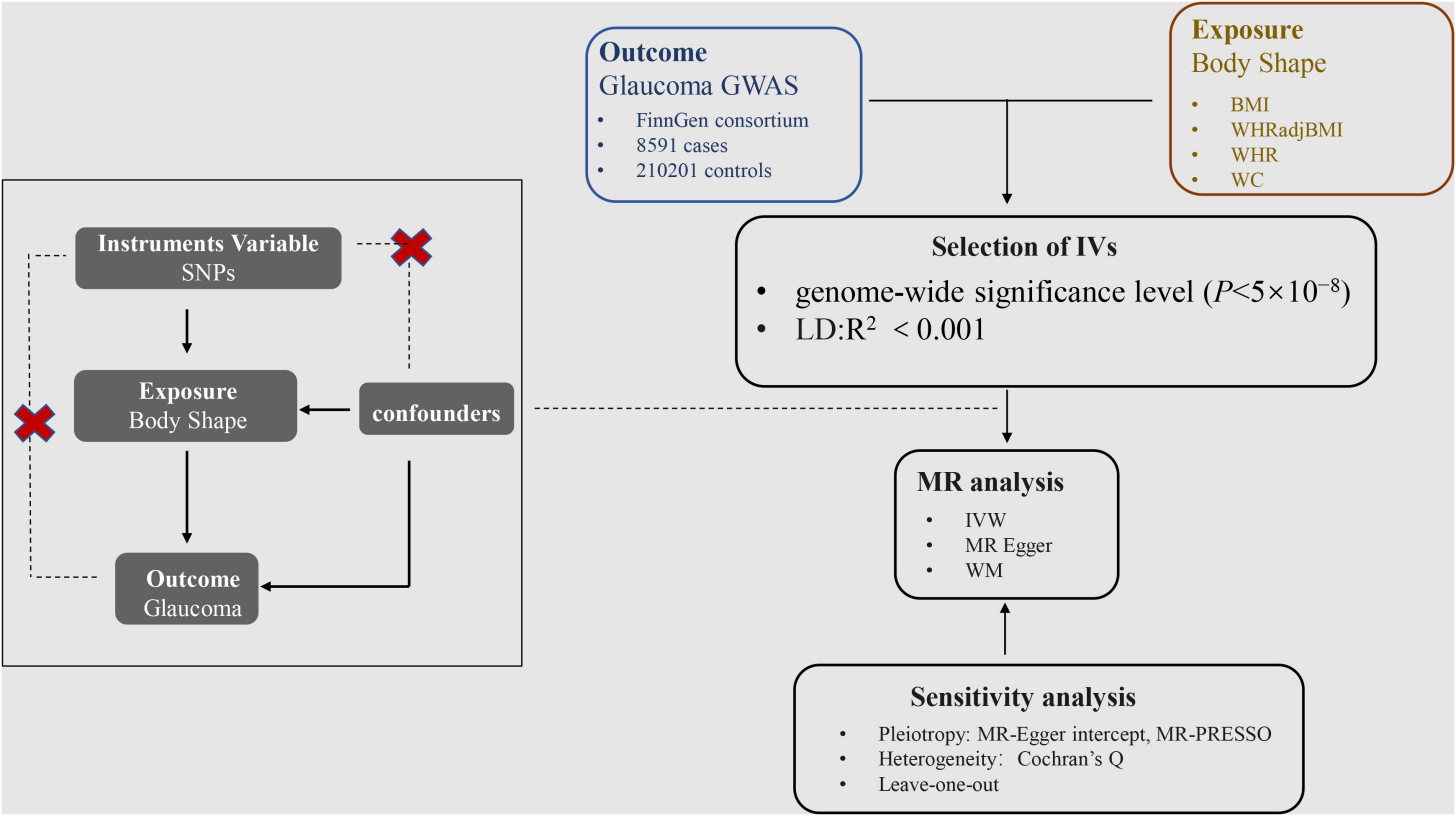
Acyclic graph interpretation of Mendelian randomization analysis.

When using a large sample size genetic database, the two sample MR analyses can assess the causal effect of BMI, WHR, WHRadjBMI, and WC on glaucoma by using SNPs as instrumental variables (IVs) to avoid accidental influence (19, 20). Our MR study was conducted on the following assumptions: First, the IVs are associated with BMI, WHR, WHRadjBMI, and WC; Second, the IVs affects glaucoma only through its effect on BMI, WHR, WHRadjBMI, and WC; Third, the IVs was not associated with any factors that confound the relationship between the exposure and outcome (Figure 1) (21). In addition, the data used by these MR analyses were processed from the data provided.

### Ethics declarations

The study used publicly available aggregate-level data without additional participant consent and ethical approval. All original and GWAS studies are approved by the ethics committees of their respective institutions. The ethical approval for each study can be found in the original publication. All methods and procedures in this study were performed in accordance with the Declaration of Helsinki. All methods in our study of MR were performed by the STROBE-MR statement (22).

### Data sources for MR analyses

The two sample MR analysis was completed utilizing summary data from genome-wide association studies (GWAS) (23). In our study, the whole subjects have homogeneous characteristics which were both from Europe. The relationships for SNPs with BMI, WHRadjBMI, WHR, and WC were derived from data aggregated from the Genetics of Anthropometric Traits (GIANT) Consortium in the year of 2015 (24).

In addition, the association of exposure relative SNPs with glaucoma was obtained from the FinnGen study (https://r5.finngen.fi/) (25). The database contains 8,591 glaucoma patients and 210,201 health participants in the European population. The demographic information of participants, such as age and gender, is provided in the FinnGen study. The data set can be accessed as required.

### The selection and validation of instrumental variables

To prove the independence of IVs, we evaluated linkage disequilibrium (LD) by testing the clustering test. Because LD can introduce bias and it was required that the instrumental variables of exposure selection should be independent of each other. This was ensured by clustering the majority of variants into a series of indexed SNPs (8). Then the SNPs were gathered by aggregating all SNPs according to LD (*R*^2^ = 0.001). The significant association between the SNPs with each SNP reaching the genome-wide significance level (*P* < 5 × 10^−8^) was also determined. Palindrome and ambiguous SNPs were discarded.

### Sensitivity analysis

To test and correct the robustness of the MR estimates, we applied sensitivity analysis. Firstly, MR-Egger's intercept values were used to assess the multiplicity of SNPs. The closer the intercept to 0, the lower the multiplicity was considered. Secondly, MR Pleiotropy RESidual Sum and Outlier (MR-PRESSO) (26) was used to evaluate further the SNPs pleiotropy that has the potential causality, and the SNPs with abnormalities were removed. When the number of variables is >50%, MR-PRESSO can still get accurate results. Meanwhile, Cochran's *Q*-test was used for the assessment of heterogeneity. Finally, to ensure the robustness of the results, the analysis was carried out using the Leave-one-out test.

### Statistical methods

All analyses were completed using R software (version 1.4.1717) and the R packages “Two Sample MR.” The Wald ratio is often accustomed to deriving causal estimates for a single SNP (8). Three MR methods, namely Inverse variance weighted (IVW) linear regression, the MR-Egger regression (27), and the Weighted median method (28), were used for overall causal estimation for multiple SNPs (29, 30). The IVW was transformed into a weighted regression of the outcome effects of instrumental variables on exposure effects to obtain an overall estimate of the impact of BMI, WHRadjBMI, WHR and WC on glaucoma risk. Meanwhile, the fixed effect model or random effect model were selected according to the heterogeneity (31, 32). The MR-Egger method can still provide unbiased estimators even if pleiotropy exists for all selected instrumental variables. Moreover, even if more than half of the instrumental variables are invalid, the Weighted median still provides a consistent estimate of the causal effect. *P* < 0.05 is considered to have potential causality.

## Results

### The causal effect of BMI on glaucoma

**Figure 2:**
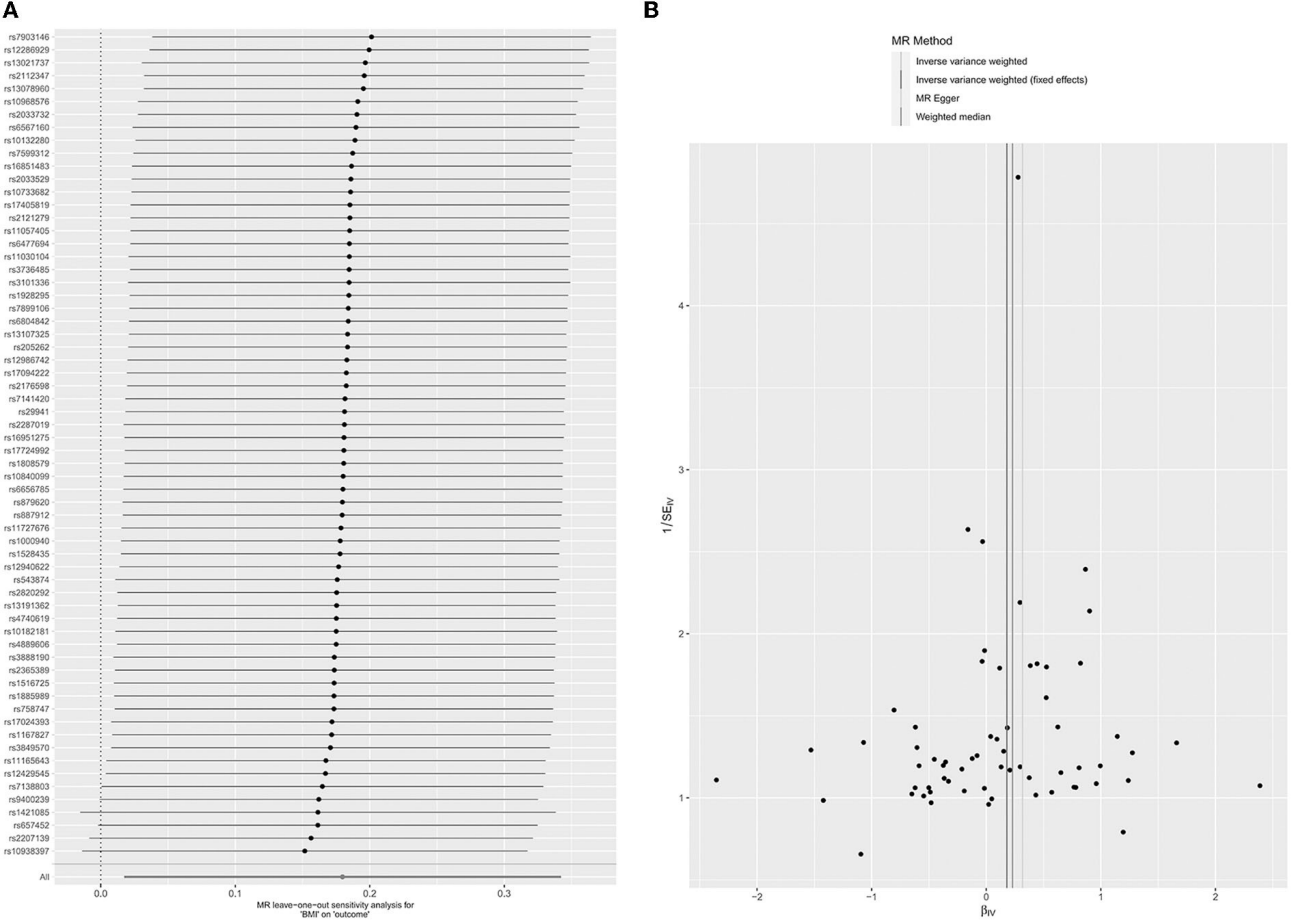
Leave-one-out permutation analysis plot **(A)** and Funnel plot **(B)** of BMI.

There were 64 SNPs associated with BMI and glaucoma (Supplementary Table 1). In MR analysis between BMI and glaucoma, overall causal estimation of the IVW method showed that BMI had a significant relationship with glaucoma (OR = 1.20; 95% CI = 1.02–1.41. *P* = 0.03) (Table 1). The Weighted median (OR = 1.26, 95% CI = 0.95–1.66; *P* = 0.11) and MR-Egger (OR = 1.37, 95% CI = 0.93–2.02, *P* = 0.12) did not show a correlation between BMI and glaucoma (Table 1; Supplementary Figure 1A). A Leave-one-out test validated the impact of each SNP on the results to verify the robustness of the data (Figure 2A). The pleiotropy were performed by MR-Egger intercept (*P* = 0.45) and MR-PRESSO (*P* = 0.63) (Table 2). At the same time, the funnel plot results confirm that there was no horizontal pleiotropy of the selected tool variables (Figure 2B), so as the results of Cochran's *Q*-test (Table 2, *P* = 0.62). As the sensitivity analysis confirms the robustness of the data and the results of the IVW were more reliable, there was a clear causal relationship between BMI and glaucoma.

**Table 1:** Associations between BS and risk of glaucoma using Mendelian randomization.

**Exposure**	**Method**	**OR**	**95% CI**	***P-*value**
BMI	IVW	1.20	1.02–1.41	0.03
BMI	Weighted median	1.26	0.95–1.66	0.11
BMI	MR Egger	1.37	0.93–2.02	0.12
WHRadjBMI	IVW	1.09	0.88–1.35	0.43
WHRadjBMI	Weighted median	1.01	0.74–1.40	0.93
WHRadjBMI	MR Egger	0.87	0.26–2.93	0.82
WHR	IVW	1.21	0.93–1.57	0.14
WHR	Weighted median	1.27	0.87–1.85	0.22
WHR	MR Egger	1.82	0.53–6.24	0.35
WC	IVW (random effects model)	1.32	1.04–1.69	003
WC	Weighted median	1.32	0.95–1.84	0.09
WC	MR egger	1.41	0.75–2.67	0.29

SNP, Single nucleotide polymorphism; BMI, including body mass index; WHRadjBMI, waist-to-hip ratio adjusted by body mass index; WHR, waist-to-hip ratio; WC, waist circumference; IVW, Inverse variance weighted; OR, The effect of the effect allele; CI, Confidence interval; *P P*-value from the GWAS.

**Table 2:** Sensitivity analysis for the associations between BS and risk of glaucoma.

**Exposure**	***P* (Cochran's Q)**	**Intercept (MR-egger)**	***P* (MR-egger)**	***P* (MR- PRESSO)**
BMI	0.62	−4.32 × 10^−3^	0.45	0.63
WHRadjBMI	0.07	6.70 × 10^−3^	0.71	0.08
WHR	0.20	−1.10 × 10^−2^	0.51	0.22
WC	0.04	−2.23 × 10^−3^	0.82	0.06

MR, Mendelian randomization; BMI, including body mass index; WHRadjBMI, waist-to-hip ratio adjusted by body mass index; WHR, waist-to-hip ratio; WC, waist circumference; SE, Standard error, *P P*-value from the GWAS.

### The causal effect of WHRadjBMI on glaucoma

**Figure 3:**
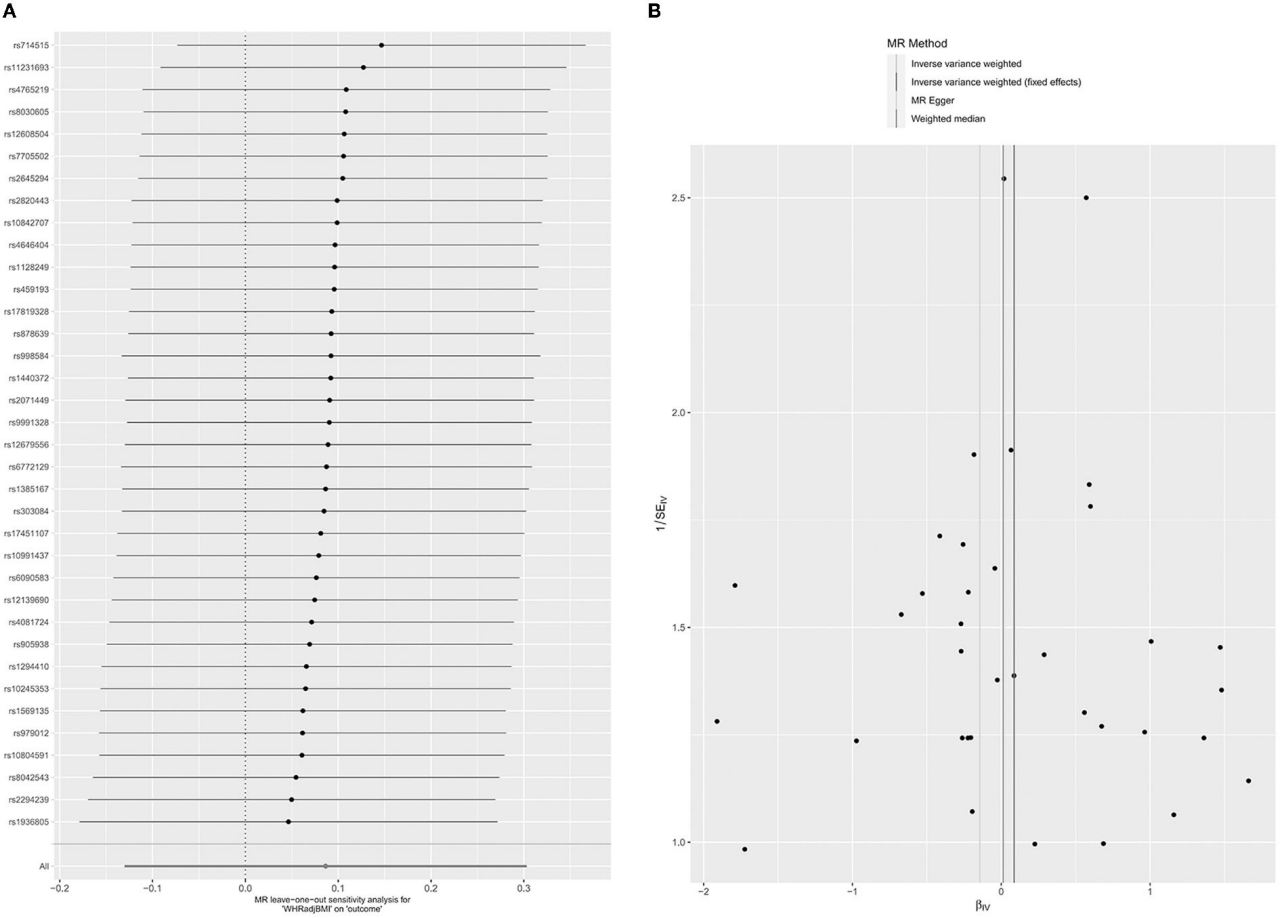
Leave-one-out permutation analysis plot **(A)** and Funnel plot **(B)** of WHRadjBMI.

Thirty-six SNPs were associated with WHRadjBMI and glaucoma (Supplementary Table 2). In the MR analysis between WHRadjBMI and glaucoma, the whole causal estimation of the IVW method showed that each SD change had no impressive impact on the risk of glaucoma in WHRadjBMI (OR = 1.10; 95% CI = 0.88–1.35; *P* = 0.43) (Table 1). The results of the weighted median (OR = 1.01, 95% CI = 0.74–1.40; *P* = 0.93) and MR egger (OR = 0.87, 95% CI = 0.26–2.93, *P* = 0.81). Did not show the correlation between WHRadjBMI and glaucoma (Table 1; Supplementary Figure 1B). The impact of each SNP on the results verified the robustness of the data by the leave-one-out of the test (Figure 3A). The pleiotropy was tested by MR egger intercept (*P* = 0.71) and MR PRESSO (*P* = 0.08) (Table 2). At the same time, the results of the funnel chart (Figure 3B) and Cochran's *Q*-test (Table 2, *P* = 0.07). Also confirmed no horizontal pleiotropy for the selected instrumental variables. Because there is no pleiotropy of the data and the results of the IVW test were more reliable, there was no obvious causal relationship between WHRadjBMI and glaucoma.

### The causal effect of WHR on glaucoma

**Figure 4:**
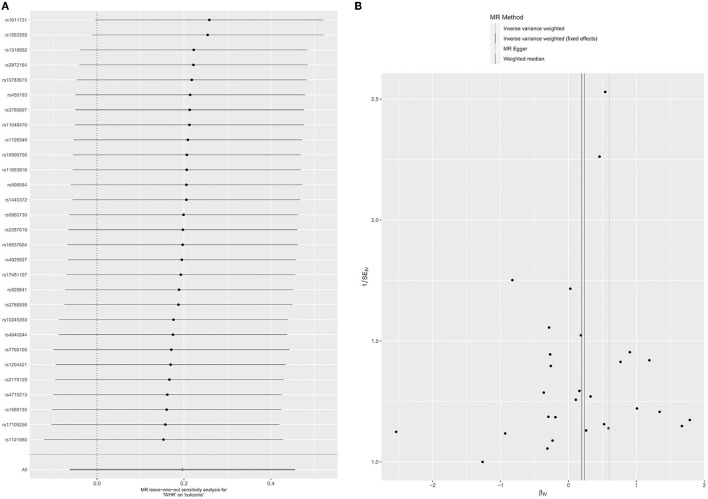
Leave-one-out permutation analysis plot **(A)** and Funnel plot **(B)** of WHR.

There were 29 SNPs associated with WHR and glaucoma (Supplementary Table 3). From the MR analyses between WHR and glaucoma, the overall causality estimated by the IVW method showed that each SD change had no significant effect on the risk of glaucoma in WHR (OR = 1.22; 95% CI = 0.94–1.58; *P*= 0.14) (Table 1). The Weighted median (OR = 1.27, 95% CI = 0.87–1.85; *P* = 0.22) and MR-Egger (OR = 1.82, 95% CI = 0.53–6.24, *P* = 0.35) did not show a correlation between WHR and glaucoma (Table 1; Supplementary Figure 1C). The impact of each SNP on the results was verified by the Leave-one-out test (Figure 4A). Pleiotropic tests were performed on MR-egger Intercept (*P* = 0.51) and MR-PRESSO (*P* = 0.22, Table 2). At the same time, the funnel plot results also confirmed that the selected instrumental variables did not have the horizontal pleiotropic effect (Figure 4B). There is no heterogeneity by using Cochran's *Q*-test (Table 2, *P* = 0.20). Since there is no pleiotropy in the data, the IVW test results were more reliable. Therefore, there was no clear causal relationship between WHR and glaucoma.

### The causal effect of WC on glaucoma

**Figure 5:**
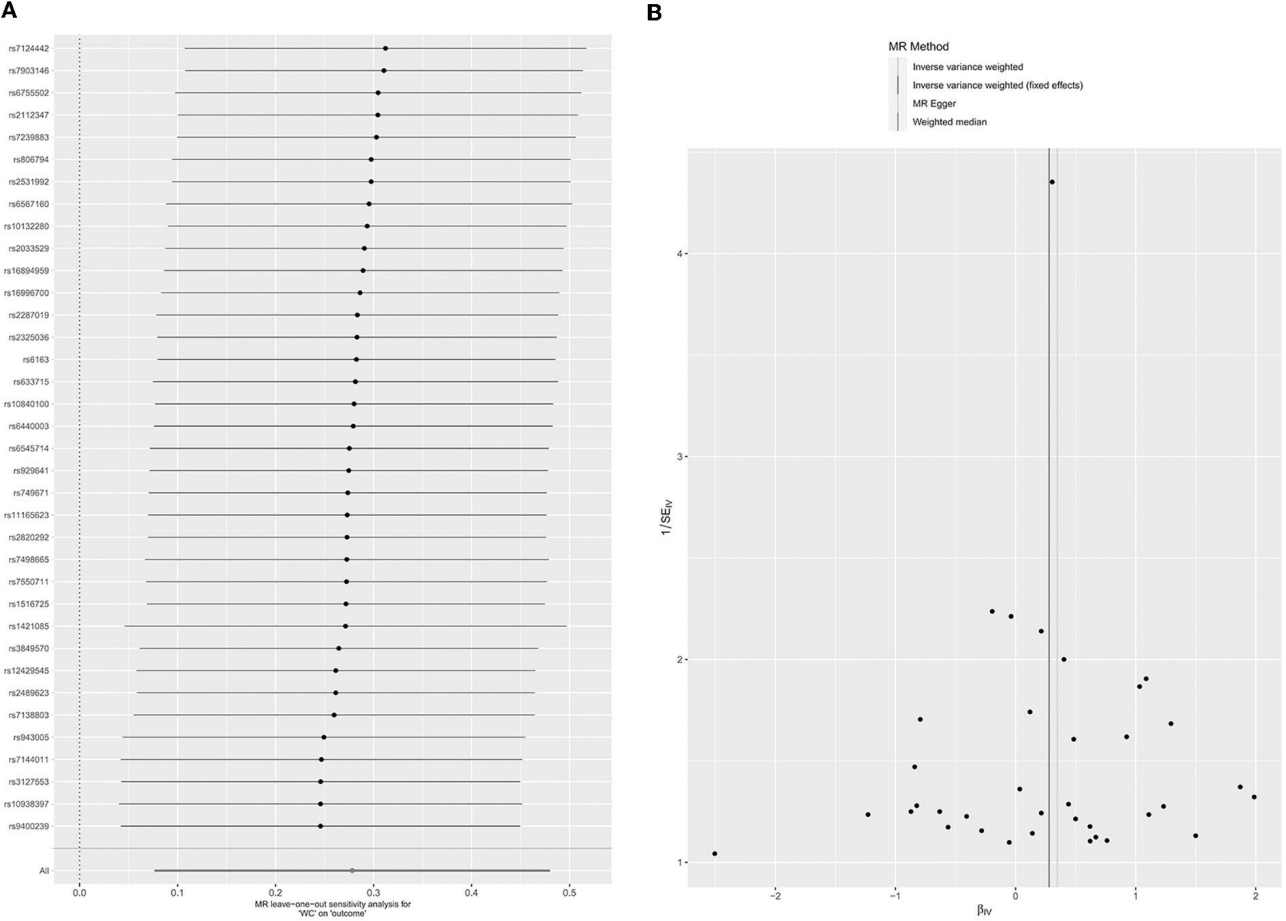
Leave-one-out permutation analysis plot **(A)** and Funnel plot of **(B)** of WC.

There were 36 SNPs associated with WC and glaucoma (Supplementary Table 4). In the MR analysis between WC and glaucoma, the overall causal estimates from the IVW method indicated that *P* = 0.01 (OR = 1.32; 95% CI = 1.08–1.62) in WC. The result of MR-Egger's sensitivity analysis (OR = 1.41, 95% CI = 0.75–2.67, *P* = 0.29) and Weighted median (OR = 1.32, 95% CI = 0.95–1.84; *P* = 0.09) in Table 1 and Supplementary Figure 1D. Pleiotropy tests were performed by MR-Egger intercept (*P* = 0.82) and MR PRESSO (*P* = 0.06, Table 2). The results of MR Egger and MR PRESSO in the pleiotropy test were >0.05 which means there was no pleiotropy. The funnel plot results also confirmed the absence of pleiotropy (Figure 5B). There is heterogeneity by using Cochran's *Q*-test (Table 2, *P* = 0.04). Considering the existence of heterogeneity, we used a random effects model (31) to IVW and we still observed a significant association between WC and glaucoma (OR = 1.32, 95 CI% = 1.34–1.69, *P* = 0.03 <0.05, Table 1). The impact of each SNP on the results was validated by a Leave-one-out test (Figure 5A). Therefore, there was an obvious causal relationship between WC and glaucoma.

## Discussion

To the best of our knowledge, this is the first MR analysis that detect the causal relationship between glaucoma and Body Size. In our study, two-sample MR analyses with a genetic instrument were selected from a wide-ranging GWAS to evaluate the body size role in the risk of glaucoma based on the genetic data obtained from European databases. MR approach is more likely to avoid confounding bias than the literature-reported risk in observational epidemiological studies (33). After adjusting for genetic linkage using the three different estimation methods (Weighted median, IVW, and MR-Egger regression), our results suggest that BMI and WC might associate with the glaucoma risk except WHRadjBMI and WHR.

In previous studies, BMI, WHRadjBMI, WHR, and WC were reported as important BS indicators. These indicators were shown to be closely related to many diseases. For instance, a cross-sectional study involving 1,891 subjects (59.1% Chinese, Malays 22.2%, Indians 18.7%) from Singapore believed that the combination of BMI and WHtR might have clinical importance for segregating patients using cardiovascular disease risk factors (34). Moreover, patients with high BMI, WC, and WHtR often represent obesity, which is considered a higher risk factor for cardiovascular disease due to abnormal blood pressure, blood lipids, blood sugar, and even blood volume (35). A study in China showed that WC and WHtR were more closely related to diabetes, especially in female patients older than 40 years old (36). This study suggested that WC and WHtR reflect the abdominal or ectopic fat in diabetes, a more important risk factor for metabolic disease than general obesity indicators. Previous studies had reported that combinations such as BMI and WHtR (Waist-to-Height Ratio) play an important role in understanding risk factors for cardiovascular disease in adults (36). Besides, somatic symptoms of depression have been considered positively correlated with anthropometric findings (38). Recently, several meta-analyses showed that different types of anthropometric data might be potential causes of gastric and esophageal cancers (39) and demonstrated the importance of anthropometric indices in disease risk factors and even disease prevention. These studies emphasized the important role of BS indicators in measuring disease risk factors.

Recently, several studies have reported BMI relationship with glaucoma. A prospective study from the Korean National Health Insurance System classified people according to metabolic syndrome and obesity, expressed the presence, severity, and concluded that metabolically healthy and obesity were had a significant risk of glaucoma. For instance, someone with a BMI of 30 kg/m^2^ or more was prone to develop glaucoma than those with a BMI of 18.5–22.9 kg/m^2^ (40). In a population-based study, Ko et al. reported that people with a BMI of 30 kg/m^2^ or higher were more likely to be associated with glaucoma incidence (41). Consistently, many studies performed on the Chinese and Singaporeans population have reported a correlation between IOP, body shapes, and BMI (15). In contrast, people with a low BMI were associated with a higher risk of glaucoma (16). These results might be influenced by the different sociodemographic groups, with differences in race, age, and population composition ratio. As another two key indicators of BS, WHR and waist circumference were documented by very few researchers (16). A study from India showed that increasing age and elevated WHR were the risk factors for elevated intraocular pressure (42). However, these cross-section studies have not completely demonstrated the relationship between BS and glaucoma (43). In our study, BMI and WC was a novel risk factor for glaucoma which is consistent with the results of previous studies (37). Hemodynamics factors have been reported to involve in the pathogenesis of POAG and metabolic health along with obesity might affect the overall primary open angle glaucoma development (7). Despite similar levels of obesity, BMI might show differential clinical outcomes, based on the body's metabolic state (44). However, BMI cannot distinguish between fat and lean body mass, while WC, WHC, etc., can reflect visceral obesity. Different BS indexes complement each other, and the synergistic effect reflects the overall body's metabolism. Jung Y pointed out that diabetes, hypertension, and hypercholesterolemia can be hypermetabolic. The metabolic syndrome may increase intraocular pressure (7) as the high blood pressure may lead to excessive aqueous humor production by facilitating more blood flow to the ciliary arteries (45). In addition, diabetes may also contribute to an increase in intraocular pressure by influencing the osmotic gradient, allowing more aqueous humor to enter the anterior chamber (46). Hyperglycemia leads to the dysfunctional trabecular meshwork, accumulation of fibronectin, disruption of water outflow facilities, changes in osmotic gradients associated with the dysregulated autonomic system, or microvascular damage to the optic nerve or peripheral retina (47). This effect might also arise due to the use of systemic drugs. However, if systemic drugs are associated with glaucoma risk, it might have implications for glaucoma prevention and treatment (31).

Few studies have explored the specific relationship between WC and glaucoma, with an Asian study of 5,255 participants from the KNHANES V database finding no relationship between WC and POAG (17), and another finding that higher WC was associated with high intraocular pressure (48). These studies have some limitations, in addition to the use of cross-sectional studies so they can't speculate on causation, they also simply studied one type of POAG in glaucoma or only studied the direct relationship between high intraocular pressure and these data. We know that although intraocular pressure is the most important factor in glaucoma, the severity of it is not directly related to intraocular pressure. Therefore, we need to find other direct factors to explain the onset of glaucoma. Our study suggests there is the potential relationship between WC and glaucoma in the Western population, and it's worth noting that people in developed countries have a propensity toward more abdominal fat (49), but it may not apply to the Chinese and most of the Asian population. So we also need to expand the database in the future. Changes in BMI and WC often represent a relative increase in fat and a relative decrease in skeletal muscle content, and according to previous reports, oxidative stress can affect glaucoma optic neuropathy (50), so we can boldly assume that it may be due to changes in body composition that cause differences in the patient's oxidative stress response and thus lead to the occurrence of glaucoma. In the life cycle, BMI is believed to increase with age (51), based on this, we can also speculate that it may be due to the influence of age on systemic metabolism, which is the phenomenon of aging and thus to indirect glaucoma, so in the future, we can refine age and even gender to the group, and further study the degree of correlation between BC and glaucoma in these different groups.

The current study had some limitations, we did not conduct a classification analysis of glaucoma, and the correlation between BS and different types of glaucoma in different regions can be further clarified in future studies. Since causality may be related to race, MR studies are required in other ethnic groups.

## Conclusion

To conclude, we performed a two-sample MR analysis. The size-related SNPs were used to study the effect of BS on the risk of glaucoma. Current research suggests increase of BMI and WC in BS might be risk factors of glaucoma. No causality was observed between WHRadjBMI and WHR and glaucoma risk. These novel findings can trigger glaucoma and can be utilized for the advancement and therapies for glaucoma in the future.

## Data availability statement

The original contributions presented in the study are included in the article/Supplementary material, further inquiries can be directed to the corresponding authors.

## Ethics statement

Ethical review and approval was not required for the study on human participants in accordance with the local legislation and institutional requirements. Written informed consent for participation was not required for this study in accordance with the national legislation and the institutional requirements.

## Author contributions

JZ and XX: conceptualization and writing—review and editing. RY: investigation and writing—original draft. KL: methodology. FH and YC: resources and supervision. All authors contributed to the article and approved the submitted version.

## Funding

This work was supported by the National Natural Science Foundation of China (No. 81600713) and Natural Science Foundation of Hunan Province (No. 2022JJ40855) to JZ and Natural Science Foundation General Program of Hunan Province (No. 2022JJ40830) and Natural Science Foundation General Program of Changsha City (No. kq2014290) to XX.

## Conflict of interest

The authors declare that the research was conducted in the absence of any commercial or financial relationships that could be construed as a potential conflict of interest.

## Publisher's note

All claims expressed in this article are solely those of the authors and do not necessarily represent those of their affiliated organizations, or those of the publisher, the editors and the reviewers. Any product that may be evaluated in this article, or claim that may be made by its manufacturer, is not guaranteed or endorsed by the publisher.

## Supplementary material

Supplementary Figure 1 Scatter plots for the genetic associations of BMI **(A)**, WHRadjBMI **(B)**, WHR **(C)**, and WC **(D)**.

## References

[1] Tham YC Li X Wong TY Quigley HA Aung T Cheng CY. Global prevalence of glaucoma and projections of glaucoma burden through 2040: a systematic review and meta-analysis. Ophthalmology. (2014) 121:2081–90. 10.1016/j.ophtha.2014.05.013 24974815

[2] Jonas JB Aung T Bourne RR Bron AM Ritch R Panda-Jonas S. Glaucoma. Lancet. (2017) 390:2183–93. 10.1016/S0140-6736(17)31469-1 28577860

[3] Liu K Xu H Jiang H Wang H Wang P Xu Y. Macular vessel density and foveal avascular zone parameters in patients after acute primary angle closure determined by OCT angiography. Sci Rep. (2020) 10:18717. 10.1038/s41598-020-73223-9 33127916 PMC7603312

[4] Tielsch JM Katz J Sommer A Quigley HA Javitt JC. Family history and risk of primary open angle glaucoma. The balti-more eye survey. Arch Ophthalmol. (1994) 112:69–73. 10.1001/archopht.1994.01090130079022 8285897

[5] Mitchell P Smith W Attebo K Healey PR. Prevalence of open-angle glaucoma in Australia. The blue mountains eye study. Ophthalmology. (1996) 103:1661–9. 10.1016/s0161-6420(96)30449-1 8874440

[6] Yu L Chen Y Xu X Dong Q Xiu W Chen Q. Alterations in peripheral B cell subsets correlate with the disease severity of human glaucoma. J Inflamm Res. (2021) 14:4827–38. 10.2147/JIR.S329084 34584441 PMC8464325

[7] Newman-Casey PA Talwar N Nan B Musch DC Stein JD. The relationship between components of metabolic syndrome and open-angle glaucoma. Ophthalmology. (2011) 118:1318–26. 10.1016/j.ophtha.2010.11.022 21481477 PMC3129406

[8] Noyce AJ Kia DA Hemani G Nicolas A Price TR De Pablo-Fernandez E. Estimating the causal influence of body mass index on risk of Parkinson disease: a Mendelian randomisation study. PLoS Med. (2017) 14:e1002314. 10.1371/journal.pmed.1002314 28609445 PMC5469450

[9] World Health Organization. Controlling the Global Obesity Epidemic. (2015). Available online at: http://www.who.int/nutrition/topics/obesity/en/ (accessed March 1, 2016).

[10] Zhang Q-Y Tie L-J Wu S-S Lv PL Huang HW Wang WQ. Overweight, obesity, and risk of age-related macular degeneration. Invest Ophthalmol Vis Sci. (2016) 57:1276–83. 10.1167/iovs.15-18637 26990164

[11] Jang H-D Kim DH Han K Ha SG Kim YH Kim JW. Relationship between intraocular pressure and parameters of obesity in Korean adults: the 2008-2010 Korea national health and nutrition examination survey. Curr Eye Res. (2015) 40:1008–17. 10.3109/02713683.2014.975367 25380054

[12] Leske MC Connell AM Wu SY Hyman LG Schachat AP. Risk factors for open-angle glaucoma. The barbados eye study. Arch Ophthalmol. (1995) 113:918–24. 10.1001/archopht.1995.01100070092031 7605285

[13] Gasser P Stümpfig D Schötzau A Ackermann-Liebrich U Flammer J. Body mass index in glaucoma. J Glaucoma. (1999) 8:8–11. 10084268

[14] Lee JY Kim TW Kim HT Lee MY Min HW Won YS. Relationship between anthropometric parameters and open angle glaucoma: the Korea national health and nutrition examination survey. PLoS ONE. (2017) 12:e0176894. 10.1371/journal.pone.0176894 28481907 PMC5421756

[15] Cohen E Kramer M Shochat T Goldberg E Garty M Krause I. Relationship between body mass index and intraocular pressure in men and women: a population-based study. J Glaucoma. (2016) 25:e509–13. 10.1097/IJG.0000000000000374 26766402

[16] Foster PJ Machin D Wong TY Ng TP Kirwan JF. Determinants of intraocular pressure and its association with glaucomatous optic neuropathy in Chinese Singaporeans: the Tanjong Pagar study. Invest Ophthalmol Vis Sci. (2003) 44:3885–91. 10.1167/iovs.03-0012 12939305

[17] Lawlor DA Harbord RM Sterne JA Timpson N Davey SG. Mendelian randomization: using genes as instruments for making causal inferences in epidemiology. Stat Med. (2008) 27:1133–63. 10.1002/sim.3034 17886233

[18] Carreras-Torres R Johansson M Gaborieau V Haycock PC Wade KH Relton CL. The role of obesity, type 2 diabetes, and metabolic factors in pancreatic cancer: a mendelian randomization study. J Natl Cancer Inst. (2017) 109:djx012. 10.1093/jnci/djx012 28954281 PMC5721813

[19] Gill D Efstathiadou A Cawood K Tzoulaki I Dehghan A. Education protects against coronary heart disease and stroke independently of cognitive function: evidence from Mendelian randomization. Int J Epidemiol. (2019) 48:1468–77. 10.1093/ije/dyz200 31562522 PMC6857750

[20] Wood A Guggenheim JA. Refractive error has minimal influence on the risk of age-related macular degeneration: a mendelian randomization study. Am J Ophthalmol. (2019) 206:87–93. 10.1016/j.ajo.2019.03.018 30905725

[21] Grover S Del GMF Stein CM Ziegler A. Mendelian randomization. Methods Mol Biol. (2017) 1666:581–628. 10.1007/978-1-4939-7274-6_29 28980266

[22] Skrivankova VW Richmond RC Woolf BAR Yarmolinsky J Davies NM Swanson SA. Strengthening the reporting of observational studies in epidemiology using Mendelian randomization: the STROBE-MR statement. JAMA. (2021) 326:1614–21. 10.1001/jama.2021.18236 34698778

[23] Zhou Y Sun X Zhou M. Body shape and Alzheimer's Disease: A mendelian randomization analysis. Front Neurosci. (2019) 13:1084. 10.3389/fnins.2019.01084 31649504 PMC6795688

[24] Xu M Li S Zhu J Luo D Song W Zhou M. Plasma lipid levels and risk of primary open angle glaucoma: a genetic study using Mendelian randomization. BMC Ophthalmol. (2020) 20:390. 10.1186/s12886-020-01661-0 33008364 PMC7532556

[25] Locke AE Kahali B Berndt SI Justice AE Pers TH Day FR. Genetic studies of body mass index yield new insights for obesity biology. Nature. (2015) 518:197–206. 10.1038/nature14177 25673413 PMC4382211

[26] Thomas DC Lawlor DA Thompson JR. Re: Estimation of bias in nongenetic observational studies using “Mendelian triangulation” by bautista et al. Ann Epidemiol. (2007) 17:511–3. 10.1016/j.annepidem.2006.12.005 17466535

[27] Haycock PC Burgess S Wade KH Bowden J Relton C Davey Smith G. Best (but oft-forgotten) practices: the design, analysis, and interpretation of Mendelian randomization studies. Am J Clin Nutr. (2016) 103:965–78. 10.3945/ajcn.115.118216 26961927 PMC4807699

[28] Bowden J Davey Smith G Haycock PC Burgess S. Consistent estimation in Mendelian randomization with some invalid instruments using a weighted median estimator. Genet Epidemiol. (2016) 40:304–14. 10.1002/gepi.21965 27061298 PMC4849733

[29] Verbanck M Chen CY Neale B Do R. Publisher Correction: Detection of widespread horizontal pleiotropy in causal relationships inferred from Mendelian randomization between complex traits and diseases. Nat Genet. (2018) 50:1196. 10.1038/s41588-018-0164-2 29967445

[30] Lawlor DA Wade K Borges MC Palmer T Bowden J. A Mendelian randomization dictionary: useful definitions and descriptions for undertaking, understanding and interpreting Mendelian randomization studies. Centre Open Sci. (2019) 10:1–109. 10.31219/osf.io/6yzs7

[31] Zhou S Zhu G Xu Y Gao R Li H Han G. Mendelian randomization study on the putative causal effects of omega-3 fatty acids on low back pain. Front Nutr. (2022) 9:819635. 10.3389/fnut.2022.819635 35237642 PMC8882682

[32] Zheng J Frysz M Kemp JP Evans DM Davey Smith G Tobias JH. Use of Mendelian randomization to examine causal inference in osteoporosis. Front Endocrinol. (2019) 10:807. 10.3389/fendo.2019.00807 31824424 PMC6882110

[33] Genomes Project Consortium A. Auton A Brooks LD DePristo MA Durbin RM. An integrated map of genetic variation from 1,092 human genomes. Nature. (2012) 491:56–65. 10.1038/nature11632 23128226 PMC3498066

[34] Emdin CA Khera AV Kathiresan S. Mendelian randomization. JAMA. (2017) 318:1925–6. 10.1001/jama.2017.17219 29164242

[35] Lam BC Koh GC Chen C Wong MT Fallows SJ. Comparison of body mass index (BMI), body adiposity index (BAI), waist circumference (WC), waist-to-hip ratio (WHR) and waist-to-height ratio (WHtR) as predictors of cardiovascular disease risk factors in an adult population in Singapore. PLoS ONE. (2015) 10:e0122985. 10.1371/journal.pone.0122985 25880905 PMC4400161

[36] Browning LM Hsieh SD Ashwell M. A systematic review of waist-to-height ratio as a screening tool for the prediction of cardiovascular disease and diabetes: 0·5 could be a suitable global boundary value. Nutr Res Rev. (2010) 23:247–69. 10.1017/S0954422410000144 20819243

[37] Zhang FL Ren JX Zhang P Jin H Qu Y Yu Y. Strong association of waist circumference (WC), body mass index (BMI), waist-to-height ratio (WHtR), and waist-to-hip ratio (WHR) with diabetes: a population-based cross-sectional study in Jilin Province, China. J Diabetes Res. (2021) 2021:8812431. 10.1155/2021/8812431 34056007 PMC8147550

[38] Wiltink J Michal M Wild PS Zwiener I Blettner M Münzel T. Associations between depression and different measures of obesity (BMI, WC, WHtR, WHR). BMC Psychiatry. (2013) 13:223. 10.1186/1471-244X-13-223 24028572 PMC3849983

[39] Du X Hidayat K Shi BM. Abdominal obesity and gastroesophageal cancer risk: systematic review and meta-analysis of prospective studies. Biosci Rep. (2017) 37:BSR20160474. 10.1042/BSR20160474 28336766 PMC5426287

[40] Jung Y Han K Park HYL Lee SH Park CK. Metabolic health, obesity, and the risk of developing open-angle glaucoma: metabolically healthy obese patients versus metabolically unhealthy but normal weight patients. Diabetes Metab J. (2020) 44:414–25. 10.4093/dmj.2019.0048 31950773 PMC7332336

[41] Ko F Boland MV Gupta P Gadkaree SK Vitale S Guallar E. Diabetes, triglyceride levels, and other risk factors for glaucoma in the national health and nutrition examination survey 2005-2008. Invest Ophthalmol Vis Sci. (2016) 57:2152–7. 10.1167/iovs.15-18373 27111561 PMC4849858

[42] Baisakhiya S Singh S Manjhi P. Correlation between age, gender, waist-hip ratio and intra ocular pressure in adult north indian population. J Clin Diagn Res. (2016) 10:CC05–8. 10.7860/JCDR/2016/21487.8991 28208848 PMC5296421

[43] Nangia V Jonas JB Matin A Bhojwani K Sinha A Kulkarni M. Prevalence and associated factors of glaucoma in rural central India. The central india eye and medical study. PLoS ONE. (2013) 8:e76434. 10.1371/journal.pone.0076434 24098790 PMC3787001

[44] Yu JH Han K Park S Lee DY Nam GE Seo JA. Effects of long-term glycemic variability on incident cardiovascular disease and mortality in subjects without diabetes: a nationwide population-based study. Medicine. (2019) 98:e16317. 10.1097/MD.0000000000016317 31335679 PMC6709246

[45] Bulpitt CJ Hodes C Everitt MG. Intraocular pressure and systemic blood pressure in the elderly. Br J Ophthalmol. (1975) 59:717–20. 10.1136/bjo.59.12.717 1218183 PMC1017441

[46] Oh SW Lee S Park C Kim DJ. Elevated intraocular pressure is associated with insulin resistance and metabolic syndrome. Diabetes Metab Res Rev. (2005) 21:434–40. 10.1002/dmrr.529 15651065

[47] Sato T Roy S. Effect of high glucose on fibronectin expression and cell proliferation in trabecular meshwork cells. Invest Ophthalmol Vis Sci. (2002) 43:170–5. 10.1109/TMAG.2008.2001677 11773028

[48] Kim HT Kim JM Kim JH Lee JH Lee MY Lee JY. Relationships between anthropometric measurements and intraocular pressure: the korea national health and nutrition examination survey. Am J Ophthalmol. (2017) 173:23–33. 10.1016/j.ajo.2016.09.031 27702621

[49] Boehm K Sun M Larcher A Blanc-Lapierre A Schiffmann J Graefen M. Waist circumference, waist-hip ratio, body mass index, and prostate cancer risk: results from the north-American case-control study prostate cancer & environment study. Urol Oncol. (2015) 33:494.e1–494.e4947. 10.1016/j.urolonc.2015.07.006 26278366

[50] Caballero M Liton PB Epstein DL Gonzalez P. Proteasome inhibition by chronic oxidative stress in human trabecular meshwork cells. Biochem Biophys Res Commun. (2003) 308:346–52. 10.1016/s0006-291x(03)01385-8 12901875

[51] Whitmer RA Gunderson EP Quesenberry Jr CP Zhou J Yaffe K. Body mass index in midlife and risk of Alzheimer disease and vascular dementia. Curr Alzheimer Res. (2007) 4:103–9. 10.2174/156720507780362047 17430231

