# Supplementary figures and images for: Body shape and risk of glaucoma: A Mendelian randomization
Source: Front Med (Lausanne). 2022 Sep 23;9:999974. doi: 10.3389/fmed.2022.999974 (PMC9538570; doi:10.3389/fmed.2022.999974)

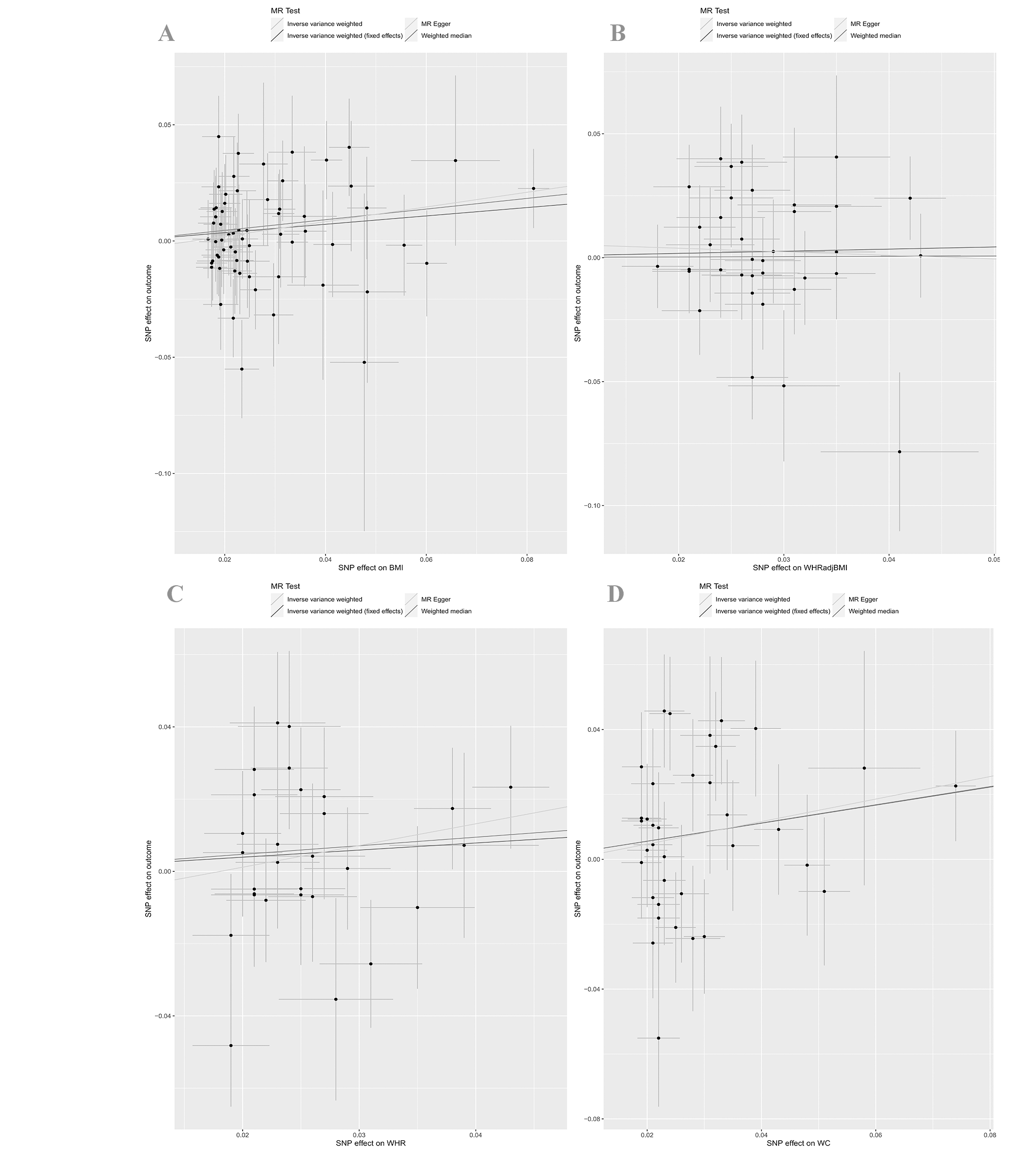

Supplement: Supplementary Figure 1 — Scatter plots for the genetic associations of BMI (A), WHRadjBMI (B), WHR (C), and WC (D). [file Image_1.TIF]
